# Adipocytokines and disease progression in endometrial cancer: a systematic review

**DOI:** 10.1007/s10555-021-10002-6

**Published:** 2021-12-24

**Authors:** Irene Ray, Lisiane B. Meira, Agnieszka Michael, Patricia E. Ellis

**Affiliations:** 1 University of Surrey, Daphne Jackson Road, Guildford, GU2 7WG UK; 2 Royal Surrey NHS Foundation Trust, Egerton Road, Guildford, GU2 7XX UK

**Keywords:** Endometrial cancer, Adipocytokines, Adipokines, Cytokines, Disease progression, Systematic review

## Abstract

The objective of the study was to document the effect of adipocytokines on endometrial cancer progression. A search of the databases CINAHL, Medline, PubMed, Cochrane, Web of Science, Embase and Google Scholar was performed for English language articles from January 2000 to December 2020 using the keywords: (Endometrial cancer) AND (progression OR metastasis) AND (adipocytokine OR adiponectin OR leptin OR visfatin OR IL-6 OR TNF-α OR adipokine OR cytokine). Forty-nine studies on adipocytokines have been included in this review. Adiponectin has been linked with anti-proliferative and anti-metastatic effects on endometrial cancer cells and is associated with a better prognosis. Leptin, visfatin and resistin are linked to the stimulation of endometrial cancer growth, proliferation, invasion and metastasis and are associated with worse prognosis or with a higher grade/stage of endometrial cancer. IL-6, Il-11, IL-31, IL-33, TNF-α, TGF-β1, SDF-1 and CXCR are involved in endometrial cancer cell growth and metastasis or involved in epithelial mesenchymal transformation (EMT) or associated with advanced disease. Adipocytokines have been found to directly impact endometrial cancer cell proliferation, invasion and migration. These molecules and their signalling pathways may be used to determine prognosis and course of the disease and may also be exploited as potential targets for cancer treatment and prevention of progression.

## **Introduction**

Rising obesity rates are thought to be an important contributor to the increasing incidence of cancer in general, including cancer of the endometrium [1]. Endometrial cancer is the 4th commonest cancer in women in the United Kingdom (UK) [2] and in the United States of America (USA) [3]. In 2018, 380,000 new endometrial cancer cases were reported worldwide [4]. In the UK, 9703 new cases were diagnosed between 2016 and 2018 [2].

There is evidence that sex differences may play a role in the prevalence of obesity-related cancers [5]. In 2014, in the USA, it was estimated that 40% of all diagnosed cancer were overweight or obesity-related, with 55% of them, being diagnosed in women compared to 24% in men [5].

Histologically, endometrial cancer is divided into two subtypes [6] — type 1 or endometrioid, which is well-differentiated, usually diagnosed at an early stage and is mostly associated with a hyper-estrogenic milieu, and type 2 endometrial cancer, which is usually of non-endometrioid, papillary-serous or clear cell subtype and is usually more aggressive in nature [7]. Although type 1 endometrial cancer accounts for approximately 80% of the cases, it is women diagnosed with type 2 endometrial cancer that have been observed to have a higher mortality rate due to the early invasion and metastasis when compared with women diagnosed with type 1 endometrial cancers [7].

Obesity is associated with a state of chronic inflammation due to the deterioration of adipose tissue functions [8, 9]. Inflamed and expanded adipose tissue associated with obesity increases cancer risk more than obesity itself [9]. Emerging scientific evidence suggests that non-cancer cells in the tumour microenvironment, such as adipocytes and macrophages, interact to enhance inflammation, leading to dysregulation of various bio-active mediators called adipocytokines and lead to re-programming of cancer cell metabolism, affecting processes involved in invasion, metastasis and immune clearance, all of which can promote tumour progression [9, 10].

Adipose tissue is the largest endocrine organ and source of adipocytokines, which is a joint term [11] used to include cytokines such as interleukin-6, 10 (IL-6,10), tumour necrosis factor-α (TNF-α), chemokine (C-C motif) ligand 2 (CCL2), transforming growth factor-β (TGF-β) and peptides called adipokines, such as adiponectin, leptin and visfatin. They are mainly but not exclusively secreted from the white adipose tissue — the adipokines from the adipocytes and the cytokines from the immune cells infiltrating the adipose tissue. They are involved in cancer signalling acting via endocrine, autocrine and paracrine routes [12]. For simplicity of description, we have discussed the adipocytokines under two sub-headings adipokines and cytokines. Adiponectin and leptin are the two most abundant adipocytokines [13].

### Adipokines

More than hundred different adipokines have been discovered. On review of the literature, adiponectin, leptin, resistin and visfatin are found to be most commonly studied in the context of endometrial cancer risk and progression [14, 15]. In obese women, adipose tissue secretes less adiponectin, which is an anti-inflammatory adipokine, and more inflammatory adipokines such as leptin and visfatin, creating an inflammatory micro-environment that stimulates a cascade of downstream inflammatory pathways that may have mitogenic, anti-apoptotic and angiogenic effects [16, 17]. Other commonly known adipokines include resistin, vaspin, omentin, chemerin, apelin, progranulin, monocyte chemotactic protein 1 (MCP-1), plasminogen activator inhibitor-1 (PAI-1), retinol binding protein-4 (RBP-4) and complement C1q tumour necrosis factor–related protein-4 (CTRP-4) [15].

Among adipocyte-derived factors, adiponectin is the most studied as it is the most abundant in the plasma [10, 18]. A review by Tumminia et al. reported that adiponectin exerts a critical role in the pathogenesis of obesity associated disorders including cancer [19]. Two meta-analyses have demonstrated decreased adiponectin levels in obese endometrial cancer patients compared to non-obese endometrial cancer patients [18, 20], from which we may infer that adiponectin, which has an anti-inflammatory effect, reduces the risk of endometrial cancer. Leptin is another commonly studied adipokine, high levels of which are associated with obesity. A recent meta-analysis [20] on endometrial cancer and adipocytokines has collected a host of evidence, all that point towards a positive association between leptin levels and endometrial cancer. Leptin directly interacts with peripheral tissues and indirectly interacts with various hypothalamic pathways that regulate immune function, cytokine production, angiogenesis, carcinogenesis and many such biological processes [10]. Leptin can activate transcriptional programs for several cellular processes, such as cell growth, proliferation, survival, migration and differentiation, all of which is commonly dysregulated in cancer [10, 21]. Another pro-inflammatory adipokine, visfatin, has been found to be significantly high in patients with endometrial cancer and has been implicated in endometrial cancer progression [22]. Similarly, another adipokine, resistin, has been reported to be involved in upregulation of inflammatory pathways and involved in the development and progression of type 2 endometrial cancer [15, 23]. Vaspin is an adipokine derived from serpin A12 (a visceral adipose-specific serpin), structurally a member of serine protease family. It has been reported to promote carcinogenesis [15]. Omentin-1 is an adipokine synthesized by vascular cells of visceral fat adipose tissue. Lower levels of circulating omentin have been associated with higher endometrial cancer incidence [24]. Omentin is considered a pro-apoptotic and anti-inflammatory adipokine [25], and several solid cancers such as colorectal, prostate and breast cancers are characterized by altered levels of omentin [26].

### Cytokines

Cytokines are low-molecular-weight proteins that mediate autocrine or paracrine signalling effects. The tumour microenvironment (TME) [27, 28] plays a pivotal role in cancer development and spread by creating a milieu where there is crosstalk between cancer cells and stromal cells. Obese adipose tissue is infiltrated by various inflammatory cells such as fibroblasts, mast cells and lymphocytes. These inflammatory cells themselves secrete or stimulate epithelial cells and pre-cancerous cells to secrete inflammatory mediators such as interleukins 6 and 11 (IL-6, IL-11) and tumour necrosis factor-alpha (TNF-α) [27]. Depending on the signalling pathways in the tumour microenvironment, the cytokines can regulate cell proliferation, survival, differentiation, migration and death. They can mount either an anti-tumoral response, as during a state of normal homeostasis, or they can also induce cell transformation (epithelial-mesenchymal transformation) and malignancy as during a state of chronic inflammation, based upon the relative concentrations of pro- and anti-inflammatory cytokines, their receptor expressions and surrounding cell activation [28]. Raised levels of IL-6 have been associated with cancer cell proliferation, angiogenesis and metastasis [29]. IL-6 stimulates adhesion and matrix molecules such as N-cadherin, vimentin, snail, twist and E cadherin and also stimulates stem cell recruitment and self-renewal which all together contribute towards not only cancer development but also its progression and distant spread [29]. A study on endometrial cancer by Chopra et al. [30] states that cytokines such as IL-8, TNF-α and TGF-β have angiogenic properties, activating various cascades of reaction involving angiogenin and other facilitatory growth factors and interleukins (such as IL-6) stimulating the proliferation of vascular endothelial cells and inducing angiogenesis which are hallmarks of cancer development. On the other hand, anti-angiogenic cytokine such as IL-10 is found to be inversely related to the levels of angiogenic cytokines.

Chemokines are a type of cytokine that induce the movement of immune cells along a chemo-attractant gradient, i.e. cells expressing a specific chemokine receptor migrate towards a higher concentration of that chemokine [31]. Chemokines potentially influence tumour infiltration, growth, cell migration and angiogenesis by facilitating movement of inflammatory and tumour cells. Chemokines such as stromal cell–derived factor 1 (SDF-1) [31] and C-C motif chemokine ligand 2 (CCL-2) have also been included in the list of adipocytokines. SDF-1 acts via receptors such as CXC chemokine receptor type 4 (CXCR4) and CXC chemokine receptor type 7 (CXCR7). CXCR4 and its ligand SDF-1 have been found to be related to chemotaxis and tumour progression and metastasis in various cancers [31].

## Objectives

The aim of this systematic review of literature is to describe the effect of adipocytokines on endometrial cancer progression. This will be achieved by reporting correlations between adipocytokines and cell proliferation, migration and invasion and/or by reporting the statistical association of adipocytokines with features of advanced endometrial cancer such as higher grade, higher stage, metastasis, reduced survival or recurrent disease.

## Methods

### Protocol and registration

A protocol for the review was devised and registered with PROSPERO (Registration No. CRD42021241605).

### Eligibility criteria

#### Inclusion criteria

- English language studies from January 2000 to December 2020
- Studies including human subjects and endometrial cancer cell lines
- Observational/ experimental study design
- Exposure of interest: adipocytokines only, i.e. any adipokine or cytokine
- Outcome of interest: endometrial cancer progression
- Effect on endometrial cancer progression demonstrated either by direct experimentation demonstrating effect of adipocytokines on cell proliferation, migration and invasion or by demonstrating correlation between adipocytokines and markers of endometrial cancer progression such as higher tumour stage, grade, myometrial invasion, lymph node invasion or distant metastasis

#### Exclusion criteria

- Review articles on the subject
- Endometrial cancer not the primary cancer
- Studies involving effect of any other biomarkers on endometrial cancer progression except for the ones mentioned in the search criteria were not included
- No mention of effect of adipocytokines on progression of endometrial cancer
- Animal studies (however, studies with both human and animal study arms have been included in the systematic review without going into any description of the animal study arm)

### Information sources

A search of the databases CINAHL, Medline, PubMed, Cochrane, Web of Science, Embase and Google Scholar was performed to identify relevant keywords contained in the title and abstract. A grey literature search was also performed to search for relevant conference abstracts, book chapters, leaflets and dissertations.

### Search strategy

The search keywords were (endometrial cancer) AND (progression OR metastasis) AND (adipocytokine OR adiponectin OR leptin OR visfatin OR IL-6 OR TNF-α OR adipokine OR cytokine). Articles published in the last 20 years (January 2000 till December 2020) in English and indexed in the above databases were identified. Studies were also identified from bibliography search of the articles found in the first search based on their title.

Thereafter, duplicate studies and animal studies that were not relevant were excluded. Full texts were obtained for articles identified by the search and considered to meet the inclusion criteria, based on their title and abstract. These articles were screened for relevance and inclusion in the systematic review for data extraction and synthesis.

The eligibility of each study was checked independently by two reviewers (IR and PE). The lists of included studies selected by the 2 reviewers were then compared, and any disagreement was resolved through discussion with an independent third reviewer (LM).

**Fig. 1:**
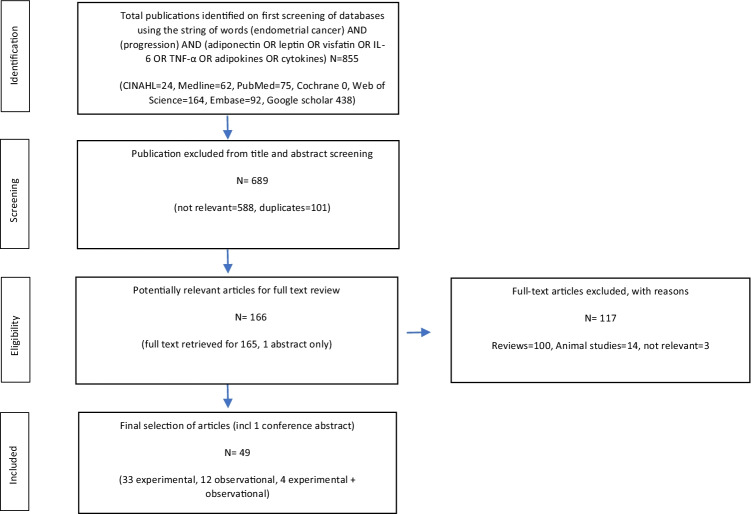
Preferred Reporting Items for Systematic Reviews and Meta Analyses Protocols (PRISMA) flow diagram of screened, excluded and analysed publications

The search for articles for the study have been performed following the PRISMA guidelines (Preferred Reporting Items for Systematic Reviews and Meta Analyses Protocols) [32] and is presented in the form of a flow-diagram (Fig. 1).

### Data extraction

Data from each study included in the review were extracted by two independent reviewers (IR and PE). Extracted data elements included first author’s name, publication year, study country, sample population — human samples/cell lines, study design and laboratory assays, primary results related to study criteria and secondary findings.

### Risk of bias in individual studies

These studies were assessed independently for their content and methodological validity by two reviewers (IR and PE), prior to inclusion in the review. Any disagreement was resolved through discussion with an independent third reviewer (LM). The studies included were assessed for their ethical conduct and sourcing of materials.

### Synthesis of results

The studies that satisfy the inclusion criteria were divided into two groups: Experimental studies and observational studies.

1. Experimental studies demonstrated the effect of adipocytokines in terms of dose-response relationships and/or time-response relationships either via cell cycle, cell invasion or cell migration studies or by demonstrating variation in the levels of various markers of cell cycle progression, cell proliferation, apoptosis, adhesion or migration in the form of dose-response and/or time-response relationships.
2. Observational studies are those that correlate the levels of adipocytokines with markers of endometrial cancer progression such as higher tumour stage or grade, more aggressive type II endometrial cancer, myometrial invasion, lymph node invasion or distant metastasis or recurrence and/or poor survival.

After dividing into the two initial groups, the findings of the studies were extracted from each study’s results and discussion sections, grouped into clusters based on their modes of action and general conclusions were drawn for each cluster in the form of narrative synthesis.

**Fig. 2:**
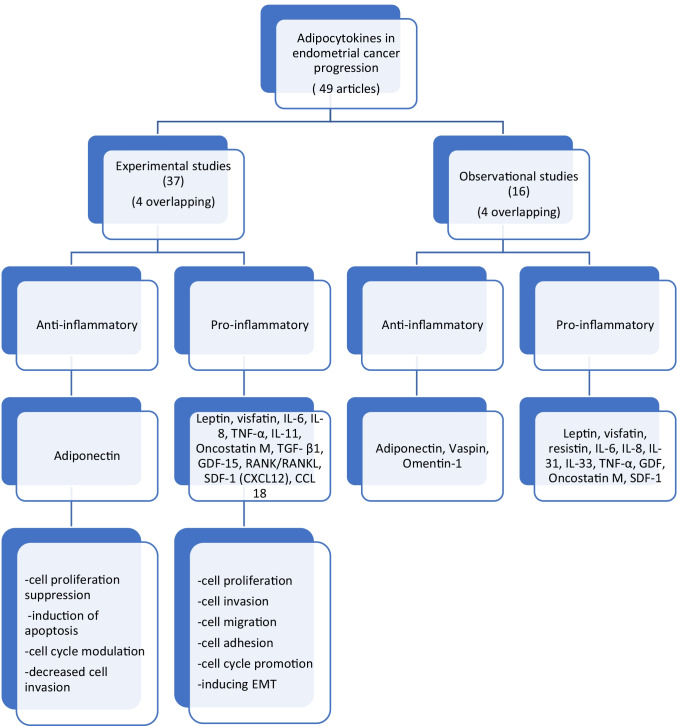
Data synthesis

The data synthesis plan has been laid out in the following flowchart (Fig. 2).

## Results

### Search results and publication characteristics

The initial database search identified 855 articles in English language that were published between January 2000 and December 2020. A total of 689 articles were excluded for duplication and lack of relevance. A full-text review was performed for the remaining 166 articles. A further 117 articles were excluded as they were either solely animal studies or review articles or not relevant as per the inclusion criteria. Finally, 49 articles have been selected for inclusion into this systematic review: 33 studies are experimental, 12 observational and 4 combined experimental and observational. The characteristics of these studies are presented in Table 1.

**Table 1:** Characteristics of review articles

**Ref No.**	**First author, year and country**	**Study population**	**Study design and laboratory methods**	**Exposure**	**Results**
[33]	Zhang (2015) China	**Human sample**: 88 endometrial cancer 90 without cancer (controls) **Cell lines**: Human endometrial cancer cell lines: AN3CA, RL95-2, Ishikawa3-H-12	**Design**: Experimental and observational **Lab methods**: 1. Proliferation assay - MTT assay 2. Apoptosis assay - Annexin V-FITC/PI	Adiponectin	1. Decreased serum adiponectin levels (<8mg/L) associated with higher grade 2/3 or lymph nodal involvement lower adiponectin <8mg/L 2. Adiponectin was reported to suppress cell proliferation and induce apoptosis in Ishiwaka cells.
[34]	Moon (2011) USA, Greece	**Cell lines**: Human endometrial cancer cell lines: RL95-2 and KLE	**Design**: Experimental and observational **Lab methods**: 1. MTT assay 2. Clonogenic assay 3. Adhesion assay 4. Matrigel invasion assay	Adiponectin	1. Adiponectin reduced cell proliferation, colony formation, adhesion, and invasion of endometrial cancer cell lines in vitro in the endometrial cancer cell lines
[35]	Cong (2007) USA, China	**Cell lines**: Human endometrial cancer cell lines: HEC-1-A and RL95–2	**Design**: Experimental **Lab methods**: 1. Flow cytometry 2. Cell cycle study 3. Apoptotic cells detection-Annexin-V-FITC kit and PI staining	Adiponectin	1. Adiponectin inhibited cell cycle proliferation as shown by an increase in cells in G0/G1 phase and a decrease in cells in in S-phase 2. Significant increase in the percentage of apoptotic cells
[36]	Wu (2006) China	**Cell lines:** Human endometrial cancer cell lines: Ishikawa cell line SPEC2 cell line	**Design**: Experimental **Lab methods**: 1. Flow cytometry 2. Proliferation assay 3. Matrigel transwell assay - cell invasion 4. Immunoblot 5. Cell immunofluorescence	Adiponectin (Acrp30) Leptin	1. Leptin treatment stimulated cell proliferation 2. Acrp30 treatment inhibited endometrial cancer cell proliferation 3. Higher apoptotic rate was noted with adiponectin and lower with leptin 4. Acrp30 increased cells in G0/G1 phase and decreased cells in S phase. Opposite effect noted with leptin. 5. Lowered invasion rate with adiponectin (66%), and increased (65%) with leptin 6. Acrp30 was able to inhibit leptin-induced SPEC-2 cell proliferation and partly suppressed the invasion stimulated by leptin
[41]	Daley-Brown (2019) USA	**Cell lines:** Human endometrial cancer cell lines: HEC-1A, Ishiwaka (Type 1 endometrial cancer) KLE, An3Ca (Type 2 endometrial cancer)	**Design**: Experimental **Lab methods**: 1. MTT assay 2. Cell cycle assay 3. Cell invasion assay 4. Annexin V - FITC/PI assay	Leptin	1. Leptin significantly increased S-phase progression. 2. Higher rate of S-phase progression noted in Type 2 endometrial cancer (2-fold increase) rather than type 1 (1.5-fold increase) 3. Higher rate of leptin induced endometrial cancer cell proliferation noted in Type 2 endometrial cancer (3-3.5-fold increase) rather than type 1 (2-2.5- fold increase) 4. Leptin also significantly induced endometrial cancer cell migration (more in type 2 endometrial cancer cells)
[71]	Zhang (2014) China	**Human sample:** 50 normal endometrium samples. 60 early endometrial cancer (stages 1 and 2) 20 late endometrial cancer (stages 3 and 4) samples	**Design:** Observational **Lab method:** 1. Immunohistochemistry	Leptin	1. Significantly higher levels of leptin and leptin receptor (ObR) in endometrial cancer tissue (72.5% and 65%) compared to normal endometrial tissue (54% and 44%). 2. Leptin concentration positively correlated with depth of myometrial invasion (p=0.001), and lymph node metastasis (not significant, p=0.156). 3. 3-year survival of leptin positive patients was significantly lower than leptin negative endometrial cancer patients (74.14% vs95.45%, p<0.05)
[66]	Zhou (2015) China	**Cell lines**: Human endometrial cancer cell lines: Ishikawa and HEC-1A	**Design**: Experimental **Lab methods**: 1. ELISA 2. Flow cytometry 3. Apoptosis assay- annexin V/PI	Leptin	1. Leptin protected endometrial cancer cells from apoptosis 2. High serum leptin concentration was correlated with degree of endometrial cancer differentiation (p = 0.035)
[70]	Cymbaluk-Płoska (2018) Poland	**Human sample:** 168 samples, endometrial cancer 92 and benign endometrium 76	**Design:** Observational **Lab methods:** 1. Multiplex immunoassay	Leptin Omentin-1 Vaspin Galectin-3	1. Higher leptin level correlated with lymph vessel involvement and poorly differentiated endometrial cancer 2. Lower concentration of Vaspin was noted in presence of LN involvement (p=0.022), lymphatic vessel invasion (p=0.03) and deep myometrial invasion (p=0.04) 3. Significantly lower concentration of Omentin-1 was noted in presence of lymphatic vessel invasion (p=0.002) and deep myometrial invasion (p=0.01). 4. Galectin -3 although found to be significantly higher in endometrial cancer patients (p=0.03), had no significant correlations with clinico-pathological features
[42]	Liu (2011) China	**Cell lines**: Human endometrial cancer cell line: Ishiwaka	**Design**: Experimental **Lab methods**: 1. Colorimetric MTT assay for cell proliferation study 2. Matrigel assay for cell invasion study	Leptin	1. Leptin stimulated the growth of Ishikawa cells in a time- and dose-dependent fashion (100ng/ml leptin) p<0.01 2. Leptin treatment (100ng/ml) also demonstrated significant endometrial cancer cell invasion p<0.01
[65]	Catalano (2009) Italy	**Cell lines**: Human endometrial cancer cell line: Ishiwaka	**Design**: Experimental **Lab methods**: 1. Flow cytometry 2. Transient transfection assay 3. Electrophoretic mobility shift assay	Leptin	1. Leptin stimulates cell cycle progression- reduced the numbers of cells in G0/G1-phase while increased cell population in S-phase (p<0.01) 2. There is also up-regulation of cyclin D1 (critical modulator of G1/S transition) together with a down-regulation of major cyclin-dependent kinase inhibitor p21^WAF1/Cip1^.
[43]	Gao (2009) China	**Cell lines**: The human endometrial cancer cell lines used were Ishikawa (36) and ECC-1, (37) both derived from well-differentiated endometrial adenocarcinoma; HEC-1A and a substrain HEC-1B derived from moderately differentiated endometrial adenocarcinoma; (38,39) RL95-2 derived from moderately differentiated adenosquamous carcinoma of the endometrium; (40) and AN3CA derived from undifferentiated endometrial adeno-carcinoma. (41) Of six cell lines, Ishikawa and AN3CA were gifts from Dr Teruhiko Tamaya (Gifu University School of Medicine, Japan); others were purchased from the America Type Culture Collection (Manassas, VA, USA). Human endometrial cancer cell lines: Ishikawa and ECC-1, HEC-1A and HEC-1B, RL95-2 AN3CA (undifferentiated endometrial adeno-carcinoma)	**Design**: Experimental **Lab methods**: 1. Thymidine incorporation assay	Leptin	1. Leptin stimulated endometrial cancer cell proliferation in all 6 endometrial cancer cell lines.
[72]	Koda (2007) Poland	**Human sample**: 60 endometrial cancer undergoing hysterectomy (different grades and stages), 25 benign, having hysterectomy for leiomyoma	**Design**: Observational **Lab methods**: 1. Immunohistochemistry	Leptin	1. No statistically significant relation between the expression of leptin (Ob), ObR, and HIF-1α with extent of tumour growth (pT), or histological grade.
[44]	Sharma (2006) USA	**Cell lines**: Human endometrial cancer cell lines: ECC1, Ishiwaka	**Design**: Experimental **Lab methods**: 1. XTT cell proliferation assay 2. Matrigel invasion assay	Leptin	1. Leptin stimulated the growth of both cell lines in a time and dose-dependent manner and the effect was maximum for 100ng/ml leptin for 24 h of treatment (p<0.01) 2. ECC1 cells exhibited remarkable invasion (p<0.01) in response to 100ng/ml leptin treatment.
[73]	Cymbaluk-Płoska (2018) Poland	**Human samples**: 64 endometrial cancer 14 non-endometrial cancer 63 normal endometrium (all had hysterectomy)	**Design**: Observational **Lab methods**: 1. Multiplex immunoassay	Visfatin	1. Significantly higher visfatin level also found in patients with invasion of blood vessels (p=0.02), lymph node metastasis (p=0.01), deeper infiltration of the endometrium (p=0.0004), (no difference in lymphatic vessel invasion) 2. The higher the visfatin level above 20.7 ng/ml, the shorter the overall survival of patients (p = 0.03)
[45]	Wang (2016) China	**Cell lines**: Human endometrial cancer cell lines: Ishiwaka, KLE cell lines	**Design**: Experimental **Lab methods**: 1. Cell proliferation assay- Cell Counting Kit (CCK)-8 2. Cell cycle analysis 3. Annexin V-fluorescein isothiocyanate (FITC) apoptosis assay	Visfatin	1. CCK 8 assay demonstrated time-dependant increased endometrial cancer cell proliferation compared with untreated control cells on exposure to visfatin (p<0.05) 2. Cell cycle analysis- reduced G1 fraction and increase in S-phase fraction 3. Visfatin treated endometrial cancer cells demonstrated decreased rate of apoptosis (p<0.05)
[74]	Ilhan (2015) Turkey	**Human samples**: 42 endometrial cancer patients and 42 controls	**Design**: Observational **Lab methods**: 1. ELISA	Visfatin Resistin	1. Visfatin had significant association with deep myometrial invasion (p=0.01) 2. Resistin was associated with increased lymph node metastasis (p=0.046)
[75]	Tian (2013) China	**Human samples**: 234 samples from endometrial cancer patients (serum-120, tissue-164, both serum and tissue- 50) Non-cancer endometrial tissues: 24 hyperplastic endometrium, 25 atypical hyperplastic endometrium, 86 normal endometrium	**Design**: Observational **Lab methods**: 1. ELISA 2. Tissue micro-array 3. Immunohistochemistry	Visfatin	1. High visfatin expression in endometrial cancer tissues was associated with advanced disease stage (p=0.023) and deep myometrial invasion (myometrial invasion ≥1/2; p=0.016). 2. Overall survival (OS) rate of endometrial cancer patients was significantly higher in the group with negative visfatin expression than with positive visfatin expression (p=0.035).
[46]	Che (2014) China	**Cell lines**: Human endometrial cancer cell lines: Ishikawa and RL95	**Design**: Experimental **Lab methods**: 1. Cell proliferation assay	IL -6	1. IL-6 increased cell growth in Ishikawa and RL95-2 cells after IL-6 treatment and the effect continued even after withdrawal of IL-6 from the medium (autocrine Il-6 signalling). 2. The concentration of IL-6 in the culture media doubled after 7 days of culture.
[47]	Che (2019) China	**Cell lines**: Human endometrial cancer cell lines: Ishikawa and RL95-2 cells	**Design**: Experimental **Lab methods**: 1. Cell proliferation assay (MTT) 2. Wound healing assay 3. Cell invasion assay (Matrigel)	IL -6 17-β estradiol	1. Administration of estradiol was found to significantly increase cells growth of endometrial cancer cells. However, treatment with IL-6-neutralizing antibody reduced the increased proliferation ability more than 60%. 2. Estradiol incubation group demonstrated faster wound healing and migration- 1.68-fold for Ishiwaka and 2.37-fold for RL95-2, which was partially attenuated, 58 and 85% respectively, by addition of IL-6 antibody
[48]	Wang (2019) China	**Human samples**: 7 paired tumour tissues and adjacent normal tissues **Cell lines**: Human endometrial cancer cell lines: Ishikawa, RL95–2, HEC1A, AN3CA, KLE cells Endometrial stromal cell (ESC)	**Design**: Experimental+ Observational **Lab methods**: 1. qRT-PCR 2. Immunoblot 3. Cell proliferation assay 4. ELISA	Yes-associated protein (YAP) IL-6 IL-11	1. YAP was upregulated in endometrial cancer cells and tissues more than ESC/benign tissue 2. Knockdown of YAP suppressed the endometrial cancer cell proliferation 3. Targeted inhibition of YAP can decrease the expression of IL-6 and IL-11 in endometrial cancer cells i.e. YAP can regulate expression of IL-6 and IL-11 in endometrial cancer cells. 4. Recombinant IL-6 or IL-11 can attenuate si-YAP suppressed proliferation of endometrial cancer cells
[49]	Chu (2018) China	**Human samples**: Human ADSCs from omentum tissues from healthy adult female donors who underwent abdominal surgery for benign gynaecologic disease	**Design:** Experimental **Lab methods**: 1. 5-Ethynyl-2′-deoxyuridine (EdU) cell proliferation kit 2. ELISA 3. qRT-PCR 4. Immunoblotting 5. IHC 6. Cell invasion assay 7. Multiplex analysis- Bio-Plex Pro Human Cytokine 17-plex Assay	IL -6 Human adipose derived stem cells (ADSC)	1. IL-6 levels were significantly increased in the supernatants of conditioned media (CM) of Ishikawa and KLE cells treated with ADSC supernatants. 2. ADSCs and IL-6 promoted the proliferation of endometrial cancer cells – 3-fold and 2-fold respectively 3. ADSCs and IL-6 promoted endometrial cancer cells invasion- 3 to 5- fold
[38]	Subramanium (2013) Malaysia	**Human samples**: Endometrial cancer tissue -4, endometrial hyperplasia- 1 **Cell lines**: 1. Human endometrial cancer cell lines: ECC-1 and HEC-1A 2. Human normal endometrial fibroblast cell line- T-HESC	**Design**: Experimental **Lab methods**: 1. Human cytokine array	IL-6, MCP-1, RANTES Cancer associated fibroblasts (CAF)	1. CAF secrete higher levels of inflammatory cytokines compared to control fibroblasts -macrophage chemoattractant protein (MCP)-1, IL-6, IL-8, RANTES
[39]	Subramanium (2016) Malaysia	**Human samples**: Endometrium formalin-fixed paraffin blocks for both benign and cancer conditions **Cell lines**: 1.Human endometrial cancer cell lines: ECC-1 and HEC-1A 2.immortalized human normal endometrial fibroblast cell line, T-HESC	**Design**: Experimental **Lab methods**: 1. Methyl thiazolyl tetrazolium (MTT) assay- cell proliferation 2. ELISA 3. Human cytokine array	IL-6 CAF	1. CAF secrete higher levels of inflammatory cytokines compared to control fibroblasts – IL-6 (10-fold), MCP-1(5.6-fold), RANTES (3-fold) 2. Endometrial cancer cell lines treated with increasing concentrations of IL-6 neutralizing antibody led to a remarkable inhibitory effect on proliferation of almost 50% in CAF conditioned media and only 5% in CAF unconditioned media. This indicates that IL-6 was present in CAFs secretion and had directly induced endometrial cancer cell proliferation. 3. Endometrial cancer cells treated with IL-6 recombinant protein without the presence of CAFs conditioned media also demonstrated dose-response increase in cell proliferation
[37]	So (2015) Korea	**Cell lines**: 1. Human endometrial cancer cell line: Ishiwaka 2. human mesenchymal stem cells (hMSCs)	**Design**: Experimental **Lab methods**: 1. Matrix metalloproteinases (MMP-2 and MMP-9) assay 1. Matrigel invasion assay 2. Wound healing assay 3. RT-PCR 4. Immunoblot 5. Human cytokine array 6. Immunofluorescence	IL-6, TGF-β1 human mesenchymal stem cells (hMSCs)	1. In the human cytokine array, only IL-6 was found to increase in all endometrial cancer and hMSC co-culture assay. 2. IL-6 and TGF-β1 treatment significantly enhance cell migration and invasion ability compared to untreated cells (p<0.05) 3. After IL-6 and TGF-β1 treatment of cancer cell lines there was decrease in an epithelial marker (E-cadherin) and an increase in mesenchymal markers (Snail, N-cadherin). 4. IL-6 and TGF-β1 treatment causes overexpression of MMP-2 and MMP-9
[50]	Che (2019)	**Cell lines**: Human endometrial cancer cell lines: Ishikawa and RL95-2	**Design**: Experimental **Lab methods**: 1. Immunofluorescence 2. Wound healing assay 3. Transwell migration assay 4. Immunoblot 5. Rt-PCR 6. ELISA	IL-6	1. IL-6 increased endometrial cancer cell migration and invasion abilities 1. IL-6 induced MMP 2 expression (EMT marker)
[76]	Kotowicz (2017) Poland	**Human samples**: 118 endometrial cancer - hysterectomy	**Design**: Observational **Lab methods**: 1. ELISA	IL 8	1. Endometrial cancer cases were found to have elevated IL-8 (65%) both in early and late stages of the disease, no correlation with stage noted. 2. Higher levels of IL -8 were associated with higher rate of relapse (p<0.011) and shorter disease-free survival (p<0.048) 3. Elevated IL-8 was found to be associated with shorter OS and it is an independent prognostic factor for OS in patients with type I endometrial cancer 4. Levels of IL-8 were significantly higher (p <0.002) in patients who died during follow-up compared to the group of surviving patients.
[77]	Smith (2012) USA	**Human samples**: 50 endometrial cancer hysterectomy samples. Among them, 26 tissue cultures were evaluated **Cell lines**: Human endometrial cancer cell lines: KLE and RL 95-2	**Design**: Observational **Lab methods**: 1. ELISA 2. Cytokine arrays	IL-8 IL-6 TNF-α	1. The highest production rates were for IL-8, which were significantly higher than rates for IL-6 which were significantly higher production rates for TNF-α 2. Rate of cytokines production was higher from primary tumour than from metastatic sites or from cell lines 3. Raised TNF-α was observed more frequently in samples from women with advanced disease (stage III/IV) 4. Marginally lower survival rates were also observed in patients with high epithelial cell IL-6 (p = 0.052) and IL-8 (p = 0.078). Paradoxically higher survival was noted in case of high stromal TNF-α (not statistically significant) 5. Combining tumour grade and cytokine levels, OS declined from 100% in patients with low-grade tumours where cytokine production rates were low, to 0% and 25%, respectively, in patients with high-grade tumours and high IL-6 production rates. Similarly, combining histology and TNF-α, the impact on survival was greater for Type 2 tumours and approached statistical significance.
[51]	Lay (2012) Australia	**Cell lines**: Human endometrial cancer cell lines: Ishikawa, HEC-1A and AN3CA (derived from endometrial cancers grade I, II and III respectively)	**Design**: Experimental **Lab methods**: 1. Cell proliferation and viability -Bromodeoxyuridine assay and WST-1 assays 2. Cell adhesion assay 3. Modified boyden chamber assay - cell migration and invasion 4. SDS PAGE 5. Immunoblot	IL-11	1. IL-11 had no effect on cell proliferation and viability 2. IL-11 increased adhesion of ANC3A cells to fibronectin, while having no effect on the other extracellular matrix proteins (co-treatment with the specific IL-11 inhibitor abolished the effect), no effect on adhesion properties of Ishiwaka and HEC1-A 3. In the AN3CA cells, IL-11 treatment resulted in a 50% increase in migration and co-treatment with the specific IL-11 inhibitor abolished the effect
[63]	Choi (2009) USA, South Korea	**Human samples**: Endometrial stromal cells derived from endometrial tissue from patient who had hysterectomy without any sign of endometrial diseases. **Cell lines**: Human endometrial cancer cell lines: HEC-1A cells (well differentiated) and KLE cells (poorly differentiated)	**Design**: Experimental **Lab methods**: 1. Matrigel invasion assay 2. Immunoblot 3. Rt-PCR 4. Immuno-precipitation 5. Wound healing assay 6. ELISA	TNF-α Hepatocyte growth factor (HGF)	1. Estrogen enhanced endometrial cancer cell invasion and progesterone inhibited it. However, addition of human recombinant TNF-α to progesterone enhanced cancer invasion (p < 0.05). 2. HGF treatment increased estradiol-induced invasiveness of endometrial cancer cells. 3. IL-6 and TNF- α are HGF inducers. Co-culture of endometrial cancer with stromal cells resulted in increasing HGF secretion dependent on increasing concentration of TNF-α. Addition of neutralising TNF- α antibody reduced estradiol mediated endometrial cancer cell invasion in both HEC 1A and KLE cell lines (p < 0.05)
[58]	Zhu (2015) China	**Human samples**: Paraffin embedded tissue blocks 73 endometrial cancer 26 normal, 20 atypical hyperplasia **Cell lines**: Human Endometrial cancer cell lines: Ishikawa and HEC-1B	**Design**: Experimental and Observational **Lab methods**: 1. IHC 2. ELISA 3. Immunoblot 4. RT-PCR 5. Cell migration and invasion assay – Transwell and Matrigel assay 6. Cell proliferation assay – CCK8 assay	Oncostatin M (OSM)	1. OSM expression higher in endometrial cancers when compared with normal endometrial tissues 2. Increased OSM expression significantly related to depth of myometrial invasion, lymph node metastasis, advanced disease stage (stages III or IV), and poor histological differentiation (grade 3) (all p<0.05). No significant difference in OSM expression between endometrioid and non-endometrioid endometrial cancer. 3. Recombinant OSM (rhOSM) promoted cell migration and invasion in Ishikawa and HEC-1B cells, however, no direct effect was found on HEC-1B or Ishikawa cell growth
[55]	Wang (2017) China	**Human samples:** Endometrial cancer tissue and adjacent normal tissue	**Design:** Experimental **Lab methods:** 1. Immunofluorescence 2. Immunoblot 3. Rt-PCR 4. ELISA 5. Matrigel invasion assay 6. Wound healing assay 7. Immunohistochemistry	Cytokines Cancer associated fibroblasts (CAF)	1. CM of CAFs had decreased levels of E-cadherin and increased the levels of N-cadherin and vimentin and showed increased invasion and metastasis in endometrial cancer cells. 2. CAF induce secretion of TGF-β 3. On addition of TGF-β, compared with the CM of CAFs, the number of invading HEC-1A and RL-952 cells were markedly increased in the CM of the normal fibroblast group, but the difference was not significant (p>0.05).
[54]	Gu (2017) China	**Human samples:** 30 endometrial cancer **Cell lines**: Human endometrial cancer cell lines: Ishikawa and AN3CA (Estrogen receptor is expressed in Ishiwaka, but not in AN3CA)	**Design:** Experimental **Lab methods:** 1. IHC 2. RT-PCR 3. Immunoblot 4. Cell proliferation assay 5. Cell migration assay	SDF-1 CXCR7	1. CXCR7 and its ligand SDF-1 were highly expressed in Ishikawa, AN3CA and endometrial cancer tissue 2. 17β- estradiol pre-treatment significantly increased the levels of CXCR7 and SDF-1 3. SDF-1 significantly promoted the growth and migration Ishikawa and AN3CA. 4. Knockdown of CXCR7 inhibited the proliferation of Ishikawa and AN3CA cells 5. Knockdown of CXCR7 inhibited 17β- estradiol and SDF-1 induced invasion in endometrial cancer cells
[78]	Walentowicz-Sadlecka(2014) Poland	**Human samples**: 92 patients with endometrial cancer had hysterectomy + archived samples	**Design:** Observational **Lab methods:** 1. IHC	SDF-1	1. SDF-1 was expressed in 90% endometrial cancer and CXCR4 and CXCR7 were found in 100% endometrial cancer compared with adjacent normal endometrial tissue. 2. Significant correlations (p<0.01) between SDF-1 and the higher clinical stage of disease, lymph node metastases, distant metastases, deep myometrial invasion (≥50%), cervical involvement, involvement of adnexa. No such correlation was found with CXCR4and CXCR7 expression 3. Significant correlation was found between SDF-1 expression and the risk of the recurrence of endometrial cancer (p = 0.0001). 4. Kaplan-Meier analyses demonstrated a stepwise reduction of OS with increasing SDF-1 expression.
[40]	Teng (2016) China	**Human samples:** 202 endometrial cancer patients 348 endometrial samples- normal endometrium, hyperplastic endometrium, atypical hyperplasia, endometrial cancer **Cell Lines:** Human endometrial cancer cell lines: HEC-1B and ECC-1 cells CAFs were isolated from endometrial tissues	**Design:** Experimental **Lab methods:** 1. ELISA 2. MTT assay 3. Transwell assay	SDF-1α, CXCR4 Macrophage chemoattractant protein-1 (MCP-1) Migration inhibitory factor (MIF) Interleukin-1 (IL-1)	1. CAFs promoted proliferation, migration, and invasion of endometrial cancer cells by secreting SDF-1α. This was blocked by AMD3100, a chemokine receptor 4 (CXCR4) antagonist. 2. CAFs secreted greater amount of SDF-1α, MCP-1, and MIF when compared to normal fibroblasts and endometrial cancer cells (SDF-1α being of the highest amount) 3. High SDF-1α expression levels were associated with deep myometrial invasion (p=0.018), lymph node metastasis (p=0.038), but not with grade or stage of endometrial cancer. Lower expression was associated with lower rate of recurrence. No such clinico-pathological connection with CXCR4.
[59]	Liu (2016) China	**Human samples:** Endometrial cancer tissue of different stages I-III) **Cell Lines:** Human endometrial cancer cell lines: HEC-1A and Ishiwaka cells	**Design:** Experimental **Lab methods:** 1. Wound healing migration assay 2. Immunohistochemistry 3. Transwell assay 4. ELISA 5. Immunofluorescence	Receptor activator of nuclear factor (RANK)/ Receptor activator of nuclear factor kB ligand (RANKL) Chemokine ligand 20 (CCL20)	1. RANK/RANKL expression was significantly elevated in higher stages of endometrial cancer 2. RANK level positively connected with N-cadherin (p = 0.0229) and vimentin (p = 0.0398), but negatively with E-cadherin (p = 0.0118) (i.e. RANK initiates EMT in endometrial cancer cells) 3. Migration and invasion of endometrial cancer cells were significantly promoted by RANK/RANKL. 4. RANK promoted expression and secretion of CCL20 5. CCL20 facilitated invasion and EMT in RANK over-expressed endometrial cancer cells
[68]	Wang (2015) China	**Human samples:** Endometrial cancer tissue of different stages I-III)- 70 samples **Cell Lines:** Human endometrial cancer cell lines: HEC-1A and Ishiwaka cells	**Design:** Experimental **Lab methods:** 1. Wound healing migration assay 2. Immunohistochemistry 3. Transwell assay 4. ELISA 5. Immunoblot	RANK/RANKL	1. High expression of RANK demonstrated decreased overall survival (p=0.01) and progression-free survival and 5-fold higher risk of death 2. RANK/RANKL significantly promoted endometrial cancer cell migration and invasion
[52]	Winship (2016) Australia	**Human samples:** Endometrial cancer tissue -10 hysterectomy Benign endometrium -4 hysterectomy **Cell Lines:** Human endometrial cancer cell lines: HEC-1A , Ishiwaka	**Design:** Experimental **Lab methods:** 1. Immunohistochemistry 2. Rt-PCR 3. PCR 4. xCELLigence real time cell proliferation assay 5. Wound healing assay	IL-11 Chondroitin sulphate proteoglycan protein (CSPG4)	1. 3-fold increase in CSPG4 gene expression after treatment with IL-11 (100 ng/ml) 2. CSPG4 protein levels are elevated in type I endometrioid cancer with increasing tumour grade G2 and G3 (p<0.05). 3. G2-derived HEC1A cells expressed CSPG4, although G1-derived Ishikawa cells did not in response to IL-11 treatment 4. CSPG4 siRNA knockdown decreases HEC1A cell proliferation and migration. 5. CSPG4 knockdown reduces SNAIL mRNA expression in HEC1A cells
[79]	Zeng (2016) China	**Human samples:** Serum samples of 160 benign, 160 endometrial cancer patients	**Design:** Observational **Lab methods:** 1. ELISA	IL-31 IL-33	1. Serum levels of IL-31 and IL-33 in patients were significantly elevated compared to those of healthy controls (p<0.0001) 2. IL-31 related to clinical characteristics, including tumor stages (p=0.024) and IL-33 related to tumour stages (p=0.035), depth of invasion (p=0.008), existence of node metastases (p=0. 029) and distant metastases (p=0.036).
[88]	Zeng (2020) China	**Human samples:** Endometrial tissue sample 150 benign, 260 endometrial cancer	**Design:** Observational **Lab methods:** 1. ELISA 2. Immunohistochemistry	IL-31 IL-33	1. IL-31, IL-33 and their receptors (IL31R and ST2) were significantly accumulated within endometrial cancer, in comparison to the controls (p<0.001) 2. Expression of IL-31 and IL-33 in endometrial adenocarcinoma tumour tissues increased with the degree of differentiation and correlated with clinical characteristics, including tumour stage, differentiation, and disease-free survival.
[60]	Wang (2014) China	**Human samples:** 70 endometrial cancer tissue samples, 30 normal endometrium, 20 endometrial hyperplasia samples **Cell lines:** Human endometrial cancer cell lines: Ishiwaka and KLE	**Design:** Experimental **Lab methods:** 1. Cell proliferation assay 2. Migration and invasion assay 3. Apoptosis analysis 4. RNA extraction 5. Rt-PCR 6. Immunoblot 7. ELISA	RANKL	1. RANK/RANKL is upregulated in endometrial cancer tissues 2. Higher RANK/RANKL expression levels were observed in carcinomas with myometrial invasion (p=0.006), lymph node metastasis (p=0.045) and lymphovascular space involvement (p=0.025) 3. RANK/RANKL promoted endometrial cancer cell proliferation, migration, and invasion
[57]	Huang (2018) China	**Human samples:** 32 paired endometrial cancer tissue and normal endometrium next to them **Cell lines:** Human endometrial stromal cell (ESC) and human endometrial cancer cell lines: HEC1A, HEC1B, ECC1, AN3CA, KLE, and RL95-2	**Design:** Experimental **Lab methods:** 1. qRT-PCR 2. Immunoblot 3. Wound healing and cell invasion assay 4. ELISA 5. Chromatin immunoprecipitation (ChIP)	TGF-β1	1. Expression of TGF-β1 in endometrial cancer tissues was significantly greater than that in the adjacent non-neoplastic normal tissues 2. Targeted inhibition of estrogen receptor (ERRα) by si-ERRα-1 suppressed migration and invasion of endometrial cancer cells and reduced expression of MMP2 and MMP-9 as well 3. si-ERRα-1 and XCT (specific inverse agonist of ERRα) can significantly decrease the expression of TGF-β1 in HEC1A cells and ECC cells 4. TGF-β1 can attenuate the XCT-790 suppressed invasion of HEC1A and ECC cells. This confirmed that ERRα regulated the motility of endometrial cancer cells via modulating TGF-β1 5. ERRα can trigger the cell migration via upregulating the expression of TGF-β1. TGF-β1 neutralization antibody can suppress the invasion of HEC1A cells.
[56]	Xiong (2016) Canada	**Cell lines:** Human endometrial cancer cell lines: KLE and HEC-50	**Design:** Experimental **Lab methods:** 1. ELISA 2. Immunoblot 3. Cell migration assay	TGF-β1	1. TGF-β1 increases the migration of type II endometrial cancer cells KLE and HEC-50
[53]	Bokhari (2015) Maryland	**Cell lines:** Human endometrial cancer cell lines: Ishikawa and HEC-1B	**Design:** Experimental **Lab methods:** 1. Cell viability assay 2. Cell invasion assay 3. Immunoblot	Chinese herbs Scutellaria baicalensis (SB) and Fritillaria cirrhosa (FC) TGF-β1	1. SB and FC treatment of cancer cells resulted in a significant decrease in expression of TGF-β isoforms 2. TGF-β1-induced cell viability and cell invasion in HEC-1B and Ishikawa cells, SB and FC inhibited this effect
[62]	Chang (2016) Taiwan	**Cell lines:** Human endometrial cancer cell lines: HEC-1A and RL95-2 cells	**Design:** Experimental **Lab methods:** 1. Cell proliferation assay 2. Cell migration assay 3. Immunoblot	Chinese herb Siegesbeckia orientalis (SOE) TGF-β1	1. TGF-β 2. 1-induced cell proliferation, cell migration, and cell invasion. SOE inhibits proliferation, migration, and invasion of endometrial cancer cells even under induction by TGF-β1. 3. TGF-β 4. 1-induces an invasive mesenchymal phenotype in endometrial cancer cells, including loss of the cell-cell junction and the formation of a lamellipodia-like structure. SOE inhibits this.
[80]	Engerud (2018) Norway	**Human sample:** endometrial cancer (235+ 466) patients Endometrial hyperplasia - 78	**Design:** Observational **Lab methods:** 1. ELISA	Plasma Growth differentiation factor-15 (GDF-15)	1. High GDF levels correlated with reduced disease-free survival (p=0.001), reduced recurrence-free survival (p<0.001), advanced FIGO stage non-endometrioid histology, high grade tumour and deep myometrial infiltration (all p<0.003), recurrent disease, lymph node metastasis, and associated with the following findings on MRI- larger tumour volume (p=0.008), deep myometrial infiltration (p=0.05) and cervical stromal invasion (=0.03)
[69]	Jing (2018) China	**Human sample:** Endometrial cancer specimen – 86 Normal endometrial tissue - 85 **Cell lines:** Human endometrial cancer cell line: Ishikawa	**Design:** Experimental **Lab methods:** 1. IHC	ER-α Chemokine Ligand 18 (CCL18)	1. M2 macrophages treated with ER-α agonist induced EMT and promoted migration of Ishiwaka cells via CCL18 and this effect was reversed by anti-CCL18 neutralising antibody
[67]	Wang (2019) China	**Cell lines:** Human endometrial cancer cell line: Ishiwaka	**Design:** Experimental **Lab methods:** 1. Immunoblot 2. Cell counting kit -8 – cell viability and proliferation assay 3. Cell scratch test – cell migration assay 4. Transwell assay	Fluorene-9-bisphenol (BHPF) TGF-β	1. BHPF significantly inhibited the EMT process of Ishikawa cells by blocking transforming growth factor-β (TGF-β) signalling pathway, more specifically by reducing the downstream proteins of TGF-β pathway, p-Smad2/3 and slug proteins 2. By the same mechanism, BHPF significantly attenuated cell viability in terms of proliferation, migration, and invasion, and eventually retarded the malignant progression of Ishikawa cells.
[61]	Schmidt (2013) Germany	**Cell lines:** Human endometrial cancer cell line: HEC-1A and Ishiwaka Osteoblast-like osteosarcoma cell line (MG63)	**Design:** Experimental **Lab methods:** 1. Microinvasion assay 2. Immunoblot	Kisspeptin 10 (KP-10) SDF-1	1. Co-culture of endometrial cancer cell with MG63 increased SDF-1 expression by MG63 2. SDF-1 induced endometrial cancer cell invasion (p<0.001) – dose dependent 3. This increased invasion rate was inhibited by co-treatment with KP-10 (p<0.001) or addition of SDF-1 antibody (p<0.05).
[64]	Billaud (2016) Abstract only		**Design:** Experimental **Lab methods:** 1. RNA sequence analysis	GDF15	1. Promotes endometrial cancer metastasis by EMT and cellular invasion

### Experimental studies

Thirty-seven articles have been included in this group (4 overlapping with observational studies). The articles have been further subdivided into pro and anti-inflammatory sub-groups alluding broadly to their role in the inflammation process and cancer progression.

#### Anti-inflammatory adipocytokines

Among the included studies, the review noted only three adipocytokines to be anti-inflammatory, such as adiponectin, vaspin and omentin-1. These anti-inflammatory adipocytokines have been found to be correlated with slowing of cancer progression. Among the anti-inflammatory adipocytokines, the mode of action of only adiponectin has been reported so far. The various mechanisms by which adiponectin suppresses cancer progression are:

- Cell proliferation suppression:

Adiponectin was shown to suppress cell proliferation in endometrial cancer cell lines in a concentration-dependent manner [33–36]. The study by Zhang et al. [33] reported a maximum reduction of cell proliferation (46.1%) with addition of 20 μg/ml adiponectin, and Wu et al. [36] demonstrated decreased proliferation of Ishikawa and SPEC -2 endometrial cancer cells by 47 and 49.5% at 20 μg/ml of adiponectin (dose-dependent decrease, *p* < 0.05).

- Induction of apoptosis:

Adiponectin was also shown to induce apoptosis in endometrial cancer cell lines in a concentration-dependent manner [33–36]. Zhang et al. [33] demonstrated a 3.45-fold increased rate of apoptosis with addition of 20 μg/ml adiponectin, and Wu et al [36] demonstrated increased rates of apoptosis (14.4%, *p* < 0.05) in Ishikawa and SPEC -2 endometrial cancer cell lines.

- Cell cycle modulation:

Wu et al. [36] also reported that adiponectin increased cells in resting cell cycle phases G1 and G0 from 48.87 to 71.14% and reduced cells in synthetic phase or S phase from 46.93 to 14.21% (both *p* < 0.05). A cancer cell is typically progressing, and hence, prevention of progression to synthetic phase will deter cancer progression.

- Decreased cell invasion:

The same study by Wu et al. mentioned above [36] also demonstrated that adiponectin decreased cell invasion by 66%. Cell invasion is a hallmark of cancer progression and hence adiponectin again demonstrated another role in prevention of cancer progression.

#### Pro-inflammatory adipocytokines

The majority of the adipocytokines reported are pro-inflammatory and act by opposing mechanisms to the anti-inflammatory adipocytokines. Included in this group are leptin, visfatin, IL-6, TNF-α, IL-8, IL-11, IL-31 and 33, TGF-β, oncostatin M, plasma growth differentiation factor-15 (GDF-15) and RANKL (receptor activator of nuclear factor kB ligand). A study by So et al. [37] culture of endometrial cancer cells produced a range of adipocytokines in the culture medium — chemokine ligand 1 (CXCL1), IL-6, IL-8, migration inhibitory factor (MIF), macrophage chemo-attractant protein-1 (MCP-1 or CCL2), Serpin E1 (PAI-1), TGF-β1 and RANTES. Cancer associated fibroblasts (CAFs) secrete about tenfold higher levels of inflammatory adipocytokines such as MCP-1, IL-6, IL-8, RANTES and SDF-1 (VEGF) than normal fibroblasts [38–40].

Several authors have studied the adipocytokines as the primary intervention in endometrial cancer progression experiments. However, we have also included in our review the studies that have demonstrated the adipocytokines to be the ‘middle-man’, i.e. acting as the mediator for other cells (such as stem cells) in endometrial cancer progression or where the adipocytokine is the point of action of some medication to stop endometrial cancer progression (such as medicinal herbs).

The pro-inflammatory adipocytokines have been found to act by the following mechanisms:

- Cell proliferation:

Leptin has been demonstrated to promote endometrial cancer cell proliferation in various studies [41–44]. Wu et al. demonstrated that leptin significantly increased the proliferation of Ishikawa cells and SPEC-2 cells by 96% and 92.5% at the concentration of 1 μg/ml and 109.5% and 103.5% at the concentration of 2 μg/ml, respectively (*p* < 0.05) [36]. Visfatin promoted cell proliferation (*p* < 0.05) in the study by Wang et al. [45].

Various studies demonstrated the proliferative role of IL-6 [37, 39, 46–50]. A study by Che et al. [47] reported that addition of IL-6 antibody to a co-culture of 17β-estradiol and endometrial cancer cells attenuates the estradiol-induced increased endometrial cancer cell growth by 60%, which leads to the conclusion that IL-6 is necessary for the estradiol-induced endometrial cancer cell growth. They also reported that estradiol promotes upregulation of IL-6 in the endometrial cancer culture medium [47]. YAP (yes-associated protein) is a transducer protein involved in the progression of various cancer types and is reported to stimulate endometrial cancer progression via IL-6 and IL-11 elevation in a study by Wang et al. [48] YAP is upregulated in endometrial cancer cell lines and tissues more than in endometrial stromal cells or benign tissue and involved in endometrial cancer progression via IL-6 and IL-11 activation. YAP induces interleukin transcription. In gene knockout experiment with small interfering-YAP (si-YAP), addition of si-YAP suppressed proliferation of endometrial cancer cells and decreased the expression of IL-6 and IL-11, which proves that YAP induces IL-6 and IL-11 which in turn promotes cell proliferation [48]. Chu et al. [49] demonstrated that IL-6 was raised in co-culture media of endometrial cancer cell and adipose-derived stem cells and that IL-6 induced endometrial cancer cell proliferation. Similarly, endometrial cancer cell lines, treated with CAF-conditioned media, lead to endometrial cancer proliferation; however, addition of increasing concentrations of IL-6 neutralizing antibody led to an inhibition of cell proliferation of almost 50% in CAF-conditioned media and only 5% in CAF-unconditioned media. This indicated that CAF stimulates endometrial cancer proliferation via IL-6 signalling [39]. Lay et al. demonstrated that IL-11 had no effect on cell proliferation [51]. A study by Winship et al. reported that IL-11 upregulated chondroitin sulphate proteoglycan (CSPG4) mRNA in endometrial cancer cell lines [52]. Silencing CSPG4 gene expression decreased endometrial cancer cell proliferation; this is an example of indirect role of IL-11 in endometrial cancer progression via CSPG4. TGF-β1 promotes endometrial cancer cell proliferation [37]. This has been indirectly demonstrated by Bokhari et al. [53] who reported that Chinese herbs *Scutellaria baicalensis* and *Fritillaria cirrhosa* are beneficial to cancer patients, and both effectively inhibit TGF-β1-induced endometrial cancer cell proliferation. SDF-1 has a role in endometrial cancer cell proliferation [40, 54]. Gu et al. [54] reported that CXCR7 and its ligand SDF-1 were highly expressed in Ishikawa, AN3CA and endometrial cancer tissue and the expression increased in response to 17β-estradiol treatment. Knockdown of CXCR7 by RNA interference (siCXCR7) inhibited the proliferation of Ishikawa and AN3CA cells. Co-culture with CAF increases SDF-1 level and promotes endometrial cancer cell proliferation [40].

- Cell migration:

IL-6 has been reported to promote endometrial cancer cell migration in various studies [37, 46, 47, 50]. TGF-β1 induces endometrial cancer cell migration including type II endometrial cancer cells [37, 55, 56].

Estrogen-related receptor alpha (ERRα) is significantly elevated in endometrial cancer tissues, especially with nodal metastasis and can directly bind to promoter of TGF-β1 and increase its transcription. ERRα can trigger endometrial cancer cell migration via regulation of TGF-β1 [57]. IL-11 treatment resulted in a 50% increase in cell migration in AN3CA endometrial cancer cells [51]. As described above, the study by Winship et al. [52] reported that IL-11 upregulated CSPG4 mRNA in endometrial cancer cell lines. Silencing CSPG4 gene expression decreased endometrial cancer cell migration. So, again, this is an example of indirect role of IL-11 in endometrial cancer progression via CSPG4. Zhu et al. [58] studied the effect of another adipocytokine Oncostatin M. Recombinant human oncostatin M promoted cell migration in endometrial cancer cell lines such as Ishikawa and HEC-1B cells. RANK/RANKL has been noted to stimulate endometrial cancer cell migration [59, 60]. Endometrial cancer cell and CAF co-culture increases SDF-1 level which in turn promotes endometrial cancer cell migration [40]. SDF-1-induced cell migration is blocked by Kisspeptin 10 (potent angiogenesis inhibitor) in the study by Shimdt et al. [61].

- Cell invasion:

Leptin demonstrates a stimulatory effect on endometrial cancer cell invasion [36, 41, 43, 44]. A study conducted by Wu et al. demonstrated a sixty five percent increased leptin-induced endometrial cancer cell invasion [36].

IL-6 and TGF-β1 treatments promote overexpression of matrix proteins such as MMP-2 and MMP-9, which are known markers for cell invasion [37, 50]. As previously described, Chu et al. [49] demonstrated that IL-6 induced endometrial cancer cell invasion when endometrial cancer cell is co-cultured with adipose-derived stem cells. TGF- β1 has been correlated with endometrial cancer cell invasion [37, 55]. As mentioned in endometrial cancer cell migration, ERRα can trigger endometrial cancer cell migration via regulation of TGF-β1 [57]. As reported in cell proliferation, Chinese herbs *Scutellaria baicalensis* and *Fritillaria cirrhosa* effectively inhibit TGF- β1-induced endometrial cancer cell invasion as also Siegesbeckia orientalis [53, 62] — this is an indirect way of demonstrating effect of TGF- β 1 on endometrial cancer cell invasion. Che et al. [47] reported that addition IL-6 antibody to a co-culture of estradiol and endometrial cancer cells abrogated the estradiol-induced increased endometrial cancer cell invasion marker MMP2 which leads to the conclusion that IL-6 is necessary for the estradiol-induced endometrial cancer cell invasion. Estradiol stimulates TNF-α expression from the endometrial cancer cells. In turn, IL-6 and TNF-α induce the stromal expression of hepatocyte growth factor (HGF) which in turn stimulates endometrial cancer invasion. Addition of hepatocyte GF (HGF) neutralizing antibody or HGF inhibitor NK4 inhibited estradiol-induced endometrial cancer cell invasion, which suggests that HGF treatment increases estradiol-induced invasiveness of endometrial cancer cells. The addition of neutralizing TNF-α antibody reduces the estradiol mediated endometrial cancer cell invasion in both HEC 1A and KLE cell lines (*p* < 0.05) which indicates that TNF-α is involved in estradiol-induced endometrial cancer cell invasion [63]. In the study by Zhu et al. above, recombinant human OSM was shown to promote invasion in endometrial cancer cell lines [58]. RANK/RANKL has been demonstrated to promote endometrial cancer cell invasion [59, 60]. A conference abstract [64] in the European journal of cancer reported that GDF15 promotes endometrial cancer metastasis by cellular invasion. SDF-1 induces endometrial cancer cell proliferation as seen in the study by Gu et al. [54], and this can be blocked by knockdown of its receptor CXCR7 by RNA interference (siCXCR7). Co-culture with CAF increases SDF-1 level and promotes endometrial cancer cell invasion [40]. This effect on invasion is also blocked by Kisspeptin 10 as in the study by Shimdt et al. [61].

- Cell adhesion:

IL-11 was also found to increase adhesion of ANC3A endometrial cancer cells to fibronectin, an extracellular matrix protein, while having no effect on the other extracellular matrix proteins [51]. In contrast to ANC3A cell line, IL-11 was found to have no effect on adhesion properties of Ishikawa and HEC1-A cell lines [51].

- Cell cycle modulation:

Leptin and Visfatin have been demonstrated to stimulate cell cycle progression to S-phase [36, 41–43, 45]. Wu et al. [36] described leptin-induced reduction of cells in G1 and G0 phase from 48.77 to 45.65% and increased cells in S-phase from 46.93 to 53.51% (both *p* < 0.05). Similar results of leptin on cell cycle promotion were noted by Catalano et al. [65] who noted an upregulation of cyclin D1, which is a critical modulator of G1/S transition alongside a downregulation of the major cyclin-dependent kinase inhibitor, p21^WAF1/Cip1^.

Che et al. [47] reported that addition IL-6 antibody to a co-culture of estradiol and endometrial cancer cells abrogated the estradiol-induced increased endometrial cancer cell cycle promoter cyclin D1 which leads to the conclusion that IL-6 is necessary for the estradiol-induced endometrial cancer cell cycle progression.

- Apoptosis prevention:

Leptin decreased the rate of endometrial cancer cell apoptosis (3.25%, *p* < 0.05) in the study by Wu et al. [36] and in a dose-dependent manner up to 150 ng/ml leptin concentration (*p* < 0.05) in the study by Zhou et al., which also demonstrated a leptin treatment-dependent reduction of active caspase-3, which is a critical effector of apoptosis in cancer [66]. Visfatin also showed similar reduction of apoptosis (*p* < 0.05) [45].

Che et al. [47] reported that addition IL-6 antibody to a co-culture of estradiol and endometrial cancer cells abrogated the estradiol-induced increased endometrial cancer cell apoptosis inhibition markers Bcl-2 and Mcl-1, which lead to the conclusion that IL-6 is necessary for the estradiol-induced inhibition of endometrial cancer cell apoptosis.

- Epithelial mesenchymal transformation (EMT):

Pro-inflammatory adipocytokines promote epithelial mesenchymal transformation, which is one of the most important steps towards cancer development and metastasis. IL-6 and TGF-β1 treatments of endometrial cancer cell lines were demonstrated to affect a decrease in epithelial marker (E cadherin) and an increase in mesenchymal markers (Twist, Snail, N-cadherin) [37, 50, 55]. Treatment with Chinese herbs *Scutellaria baicalensis* and *Fritillaria cirrhosa* in the study by Bokhari et al. [53] and *Siegesbeckia orientalis* in the study by Chang et al. [62] and fluorene-9-bisphenol in the study by Wang et al. [67] inhibited TGF-β1-induced expression of EMT markers Snail, Slug and Focal adhesion kinase, again demonstrating, indirectly, the effect of TGF-β1 on EMT. A conference abstract [64] in the European journal of Cancer reported that GDF15 promotes endometrial cancer metastasis by EMT. RANK level has been positively correlated with N-cadherin (*p* = 0.0229) and Vimentin (*p* = 0.0398), but negatively with E-cadherin (*p* = 0.0118), indicating its role in EMT. RANK overexpressed endometrial cancer cells had higher levels of CCL20 which facilitates invasion and EMT of RANK overexpressed endometrial cancer cells [68]. CCL-18 has been shown to have a mediating effect on endometrial cancer progression by EMT in a study by Jing et al. [69].

The roles of adipocytokines mentioned above have been summarized in a table format (Table 2).

**Table 2:** Role of adipocytokines in endometrial cancer progression

	Adipocytokine	Mode of action in cancer progression
Anti-inflammatory	Adiponectin	Cell proliferation suppression [33–36] Induction of apoptosis [33–36] Cell cycle modulation [36] Decreased cell invasion [36]
Pro-inflammatory	Leptin	Promotes cell proliferation [41–44] Promotes cell invasion [36, 41–44] Cell cycle modulation [36] Prevention of apoptosis [36, 66]
	Visfatin	Promotes cell proliferation [45] Cell cycle modulation [45] Prevention of apoptosis [45]
	IL-6	Promotes cell proliferation [37, 39, 46–50] Promotes cell migration [37, 46, 47, 50] Promotes cell invasion [37, 46, 47, 49, 50] Cell cycle modulation [47] Prevention of apoptosis [47] Epithelial mesenchymal transformation [37, 50, 55]
	IL-11	Promotes cell proliferation [48, 52] Promotes cell migration [51, 52] Promotes cell adhesion [51]
	TNF-α	Promotes cell invasion [63]
	Oncostatin-M	Promotes cell invasion [58]
	TGF-β1	Promotes cell proliferation [37, 53] Promotes cell migration [37, 55–57] Promotes cell invasion [37, 53, 55, 57, 62] Epithelial mesenchymal transformation [37, 50, 53, 55, 62, 67]
	Plasma growth differentiation factor (GDF-15)	Promotes cell invasion [64] Epithelial mesenchymal transformation [64]
	Receptor activator of nuclear factor kB ligand (RANKL)	Promotes cell proliferation [60] Promotes cell migration [59, 60] Promotes cell invasion [59, 60] Epithelial mesenchymal transformation [68]
	SDF-1/ CXCR	Promotes cell proliferation [40, 54] Promotes cell migration [40, 61] Promotes cell invasion [40, 54, 61]
	CCL-18	Epithelial mesenchymal transformation [69]

### Observational studies

Sixteen articles have been included in this group (4 overlapping with experimental studies).

#### Anti-inflammatory adipocytokines

As mentioned in experimental studies, adiponectin is the main anti-inflammatory adipocytokine. Lower levels of anti-inflammatory adipocytokines have been associated with progression of endometrial cancer such as higher grade, stage and lymph node involvement.

Low levels of adiponectin (<8 mg/l) were found to have significant association with higher stage II or III, grade 3 and lymph node involvement, which are clinico-pathological markers for endometrial cancer progression [33]. There was a paucity of studies on other anti-inflammatory adiponectin, but in an observational study by Cymbaluk-Płoska et al. [70], lower concentration of Vaspin was noted in presence of LN involvement (*p* = 0.022), lymphatic vessel invasion (*p* = 0.03) and deep myometrial invasion (*p* = 0.04) and significantly lower concentration of Omentin-1 in the presence of lymphatic vessel invasion (*p* = 0.002) and deep myometrial invasion (*p* = 0.01) [70].

#### Pro-inflammatory adipocytokines

Higher levels of pro-inflammatory adipocytokines such as leptin, visfatin, resistin, IL-6, IL-8, IL-31, IL-33, TNF-α, GDF, Oncostatin M and SDF-1 are associated with an advanced state of endometrial cancer such as high grade II/III, deeper myometrial invasion, lymph vessel invasion, lymph node metastasis, shorter survival and recurrence [40, 58, 59, 66, 68, 70–80].

Leptin levels have been found to be positively correlated with depth of myometrial invasion, lymph node metastasis [71], lymph vessel involvement [70], poor 3-year survival [71] and poorly differentiated endometrial cancer [66, 70] which indicate an advanced disease but was found to be inversely related with histological grade [71]. Koda et al. [72] also observed correlation between positive leptin expression and more locally advanced and moderately differentiated endometrial cancer, although the results did not reach statistical significance. Significantly higher concentrations of visfatin have been demonstrated [73] in patients with advanced endometrial cancers, for example in cases with invasion of blood vessels, lymph node metastasis, deeper infiltration of the endometrium [73] or deeper myometrial invasion [74, 75]. Mean visfatin levels were significantly different between depth of myometrial invasion (10.6 ± 7.6 ng/ml in <50% invasion and 23.5 ± 16.2 ng/ml in >50% invasion, *p* = 0.019) [74]. No difference was found in case of lymphatic vessel invasion [73]. Setting a cut-off level for visfatin at 20.7 ng/ml, the higher the levels of visfatin, the shorter was the overall survival of patients (*p* = 0.03) [73]. Similar results in the study by Tian et al [75], where overall survival rate of endometrial cancer patients was significantly higher in the group with negative visfatin expression than with positive visfatin expression (*p* = 0.035). Resistin was associated with increased lymph node metastasis (*p* = 0.046) in the observational study by Ilhan et al. [74].

IL-8 concentration was found to be 65% elevated in patients with endometrial cancer compared to control population (*p* < 0.0001). However, no correlation with stage or grade of endometrial cancer was noted [76]. In the study by Smith et al. [77], IL-8 levels were significantly higher than IL-6 levels, which were significantly higher than production rates for TNF-α in endometrial cancer cells. Cytokines were more abundant in cells derived from primary tumours than from metastatic sites (*p* < 0.001 for IL-6) or endometrial cancer cell lines (*p* < 0.001 for IL-6) [77]. Patients who relapsed had higher levels of IL-8 (*p* < 0.011) and were associated with shorter disease-free survival (*p* < 0.048) and overall survival. Levels of IL-8 were significantly (*p* < 0.002) higher in patients who died during follow-up [76]. Smith et al. [77] observed epithelial TNF-α to be more abundant in endometrial cancer tissue samples from women with advanced stages of endometrial cancer (stage III/IV) (*p* = 0.018) and was associated with lower survival rate (*p* = 0.032). Marginally lower rates of survival were also observed in patients with high epithelial cell IL-6 (*p* = 0.052) and IL-8 (*p* = 0.078) [77]. Poor overall survival was noted in tumours with high cytokine concentration such as high IL-6 and TNF-α. IL-31 and IL-33 were significantly accumulated in cancer patients (*p* < 0.001) and higher levels correlated with higher grade of endometrial cancer (*p* < 0.001) which shows that IL-31 and 33 are associated with a more severe or progressed disease [79]. Serum IL-31 was related to tumour stages (*p* = 0.024), and serum IL-33 was related to tumour stages (*p* = 0.035), depth of invasion (*p* = 0.008), nodal metastases (*p* = 0.029) and distant metastases (*p* = 0.036) [79]. Their receptors (IL-31R and ST2 respectively) were also highly expressed in endometrial cancer cells (*p* < 0.001) and higher expression correlated with advanced endometrial cancer and with shorter disease-free survival (all statistically significant) [79]. Only one study by Zhu et al. [58] has been included which reported high oncostatin M expression in endometrial cancer tissue compared to non-cancer endometrial tissue. Significant association was found between increased oncostatin M expression and the depth of myometrial invasion, lymph node metastasis, advanced disease stage (stages III or IV) and poor histological differentiation (grade 3). Engerud et al. [80] noted that high GDF levels correlated with severe disease and independently predicted recurrent disease (OR = 3.14; 95% CI 2.10–4.76) and lymph node metastases (OR = 2.64; 95% CI 1.52–4.61). Patients with high plasma level of GDF-15 had significantly larger tumour volume (*p* = 0.008). Preoperative plasma level of GDF-15 was significantly higher for patients who experienced recurrence than those who did not develop recurrence and the levels at the time of recurrence were greater than at baseline (*p* = 0.001). High GDF levels correlated with reduced disease-free survival (*p* = 0.001); reduced recurrence-free survival (*p* < 0.001); advanced FIGO stage non-endometrioid histology, high-grade tumour and deep myometrial infiltration (all *p* < 0.003); recurrent disease, lymph node metastasis and associated with larger tumour volume (*p* = 0.008); deep myometrial infiltration (*p* = 0.05); and cervical stromal invasion (*p* = 0.03) on MRI imaging. RANK/RANKL expression was significantly elevated in endometrial cancer tissue of higher stage, i.e. deeper myometrial invasion, LN and vascular involvement, and showed decreased progression-free survival and overall survival (5 fold higher risk of death) [59, 60, 68]. In a study by Walentowicz-Sadlecka et al. [78], SDF-1 was expressed in 90% of endometrial cancer tissue samples and CXCR4 and CXCR7 were found in 100% endometrial cancer tissue samples. Adjacent normal tissue showed very little expression of CXCR4 and no expression of SDF-1 or CXCR7. Statistically significant correlations were found between SDF-1 and higher clinical stage of endometrial cancer, lymph node metastases, distant metastases, deep myometrial invasion (≥50%), cervical involvement and involvement of adnexa. SDF-1 expression was significantly correlated with the risk of the recurrence (*p* = 0.0001). Overall survival showed stepwise impairment with increasing SDF-1 expression by Kaplan-Meier analyses. In another study by Teng et al. [40], high SDF-1α expression levels were associated with deep myometrial invasion (*p* = 0.018), lymph node metastasis (*p* = 0.038), poor survival (*p* = 0.039) and higher recurrence rate (*p* = 0.032).

### Risk of bias in individual studies

Each study was evaluated for their methodological integrity, ethical sourcing of materials and conduct of study before including in the review. However, it was not possible to evaluate the quality of conference abstracts which were included in the review as a part of grey literature search to increase the coverage of the review.

### Risk of bias across studies

We attempted to reduce publication bias by including grey literature search. However, our specified (2000–2020) timed search may have some introduced a time-period bias and English-language literature search may have introduced a language bias.

### Strengths and limitations of our review

The strength of our review is that it presents a relatively comprehensive review of all English language studies in the last 20 years demonstrating the effect of adipocytokines on endometrial cancer progression, their association with poor clinic-pathological markers and their pathway of action. However, use of a specified time range and English language for our search criteria may have introduced a time-period bias and a language bias, respectively. We have performed a grey literature search to reduce the risk of selection bias and have included one conference abstract. PRISMA flowchart has been used to reduce publication and selection bias. All the studies included used either human tissue or endometrial cancer cell lines, mentioned the source of all ingredients and described in detail the tests involved such as proliferation, invasion assay and migration assay. The studies included are from different parts of the world, using cell lines or human tissue from varying sources across the world, but similar results have been found in majority of the studies.

The limitations include that the articles included are not uniform in their cell type/ tissue testing, various studies have used various cell lines procured from various places. Some studies have used human tissue, some fresh, some frozen, or some have used endometrial cell lines and some both. They have also used different tests and ingredients to check for cell proliferation, invasion, or migration. No statistical tests have been used.

## Further recommendations for research

The current review shows the various ways in which adipocytokines can affect endometrial cancer progression. Further research should explore the signalling pathways by which these adipocytokines affect tumour progression. The inflammatory pathways predominantly reported in the literature on progression of endometrial cancer are the MAPK, JAK/STAT3 and PI3K/AKT /mTOR pathways [81, 82]. Studies are in progress on pI3K/Akt/m-TOR pathway being treated as a therapeutic target in endometrial carcinoma with m-TOR, PI3K, dual PI3K/m-TOR and Akt inhibitors [83, 84], and also, inhibition of the IL-6 receptor and its downstream effectors JAK1 (ruxolitinib) and STAT3 (nifuroxazide) are being investigated for its effect on tumour cell growth *in vivo* and *in vitro* studies [85]. mTORC1 inhibitors currently in clinical development stage are everolimus, temsirolimus and ridaforolimus, which have all showed antitumor activity in endometrial cancer cell lines [86]. In a study by Cantrell et al., metformin has been reported to inhibit endometrial cancer cell proliferation, which was partially mediated through AMPK activation and subsequent inhibition of the mTOR pathway [87]. Further trials should be directed towards developing adipocytokine-targeted therapy, which will involve specific inhibitors at various stages in their signalling pathways, which may block the proliferation, invasion and migration of endometrial cancer cells and therefore reduce tumour invasion and metastasis in endometrial cancer.

## Conclusion

Various adipocytokines have been implicated in the pathogenesis of endometrial cancer by their involvement in the invasion and migration of the cancer cells. This may lead to a higher stage of disease, which ultimately culminates in a poor prognosis, poor response to anti-cancer treatment, recurrence, reduced disease-free survival or reduced overall survival. Defining the biomarkers involved can contribute to a better tailoring of endometrial cancer treatment to target specific markers and their downstream effectors which would reduce the risk of progression or recurrence of cancer. To the best of our knowledge, this study is the only systematic review on the effect of adipocytokines on endometrial cancer progression and summarises all the relevant English language articles on this subject in the last 20 years. Only a few adipocytokines have been found to be anti-inflammatory in our review such as adiponectin, vaspin and omentin-1, and they have been associated with better prognostic factors such as lower stage and grade of disease. Adiponectin has also been found to slow cancer progression by cell proliferation suppression, induction of apoptosis, cell cycle modulation and decreased cell invasion. On the other hand, there have been studies on several pro-inflammatory adipocytokines such as leptin, visfatin, resistin, IL-6, IL-8, IL-31, IL-33, TNF-α, GDF, oncostatin M and SDF-1, which are not only associated with a more advanced endometrial cancer disease state and poor prognostic factors, but they also promote endometrial cancer progression by mechanisms such as cell proliferation, invasion, migration, adhesion, cell cycle promotion and by inducing EMT. However, the studies are not extensive and need further investigation to confirm the mechanism of action of these adipocytokines and their inter-relationships. More data is needed to create a prediction model for prognostication, identifying patients at highest risk of disease progression and hence suitable for adjuvant chemotherapy and for remission monitoring. Larger prospective studies are needed to assess the effect of the various adipocytokines on endometrial cancer progression in the same cohort of patients or the same endometrial cell lines to provide a uniform basis of comparison across studies for a meta-analysis. Some of the articles in our review demonstrated that adipocytokine-specific inhibitors abrogated endometrial cancer cell proliferation, invasion and migration, so we can infer that possibly adipocytokine-targeted therapy could not only treat cancer but also prevent cancer invasion and metastasis. Targeted treatment drugs trials should probably be the next step in prevention and control of this rapidly increasing gynaecological cancer.
